# CRISPR/Cas9‐mediated editing of 1‐aminocyclopropane‐1‐carboxylate oxidase1 enhances *Petunia* flower longevity

**DOI:** 10.1111/pbi.13197

**Published:** 2019-07-02

**Authors:** Junping Xu, Beum‐Chang Kang, Aung Htay Naing, Su‐Ji Bae, Jin‐Soo Kim, Hyeran Kim, Chang Kil Kim

**Affiliations:** ^1^ Department of Horticultural Science Kyungpook National University Daegu Korea; ^2^ Center for Genome Engineering Institute for Basic Science Daejeon Korea; ^3^ Department of Biological Sciences Kangwon National University Chuncheon Korea

**Keywords:** ethylene production, gene expression, genome editing, mutation, *Petunia hybrida*

## Abstract

The genes that encode the ethylene biosynthesis enzyme 1‐aminocyclopropane‐1‐carboxylate oxidase (ACO) are thought to be involved in flower senescence. Hence, we investigated whether the transcript levels of *PhACO* genes (*PhACO1*,* PhACO3* and *PhACO4*) in *Petunia* cv. Mirage Rose are associated with ethylene production at different flowering stages. High transcript levels were detected in the late flowering stage and linked to high ethylene levels. *PhACO1* was subsequently edited using the CRISPR/Cas9 system, and its role in ethylene production was investigated. *PhACO1*‐edited T_0_ mutant lines, regardless of mutant type (homozygous or monoallelic), exhibited significantly reduced ethylene production and enhanced flower longevity compared with wild‐type. Flower longevity and the reduction in ethylene production were observed to be stronger in homozygous plants than in their monoallelic counterparts. Additionally, the transmission of the edited gene to the T_1_ (lines 6 and 36) generation was also confirmed, with the results for flower longevity and ethylene production proving to be identical to those of the T_0_ mutant lines. Overall, this study increases the understanding of the role of *PhACO1* in petunia flower longevity and also points to the CRISPR/Cas9 system being a powerful tool in the improvement of floricultural quality.

## Introduction

Petunias, widely used as a bedding plant in the floricultural industry, have become increasingly popular due to their diversity of different flower shapes and colours. Generally, petunias exhibit excellent flower longevity because they continuously produce new flowers over an extended period. However, newly produced individual flowers exhibit rapid senescence in the mother plants. Because petunias are ethylene‐sensitive, their flower senescence is associated with an increase in ethylene production (Woltering and Van Doorn, 1988; Tang *et al*., 1993; Tang and Woodson, 1996; Huang *et al*., 2007). Hence, it is necessary to find an effective way to reduce ethylene production.

Ethylene is derived from methionine, which is converted to *S*‐adenosylmethionine (SAM or AdoMet) by SAM synthetase and then to 1‐aminocyclopropane‐1‐carboxylic acid (ACC) by ACC synthetase, before ACC oxidase (ACO) finally converts ACC to ethylene (Yang and Hoffman, 1984). In petunias, it has been reported that *PhACO1*,* PhACO3* and *PhACO4* encode ACO and are expressed in the petals and pistils during flower development (Huang *et al*., 2007; Tang and Woodson, 1996; Tang *et al*., 1993). However, the functional roles of *PhACO* genes have not yet been closely investigated using reverse and forward genetics.

Huang *et al*. (2007) reported that the transformation of antisense *BoACO1* genes in broccoli, whose sequences were approximately 90% homologous to those of *PhACO* in petunia, resulted in the reduction in ethylene production and delayed flower senescence. Alternative approaches to suppressing ethylene levels suffer various limitations. For example, although ethylene biosynthesis in cut flowers can be blocked by adding chemicals such as sodium nitroprusside and nano‐silver to vase water (Naing *et al*., 2017a,2017b,2017c), this is not effective for a bedding plant like petunias (Porat *et al*., 1993). It is also difficult to obtain desirable traits using classical breeding techniques because the introduction of the desired gene is imprecise and uncertain. Moreover, the application of RNA interference (RNAi) and virus‐induced gene silencing (VIGS) methods to suppress the transcription level of the genes associated with ethylene biosynthesis is only promising for high‐throughput analysis and temporal purposes (Reid *et al*., 2009). Therefore, the editing or deletion of the ethylene biosynthesis genes at the genome level is more promising than the above‐mentioned methods for the permanent reduction in ethylene production.

The deletion or editing of target genes can be achieved using three genome editing methods: zinc‐finger nucleases (ZFNs), transcription activator‐like effector nucleases (TALENs) and clustered regulatory interspaced short palindromic repeat (CRISPR)/Cas9) sequences. Of these, the CRISPR/Cas9 system has received significant recent attention because of its high specificity for the editing of target genes, its low cost and the simplicity of its design (Cong *et al*., 2013; Feng *et al*., 2013; Shan *et al*., 2013). This system induces site‐specific double‐strand breaks (DSBs) at a target site upstream of the protospacer‐adjacent motif sequence (PAM) within the genome, followed by a DSB repair mechanism consisting of homologous recombination (HR) or non‐homologous end‐joining (NHEJ). HR leads to the accurate reconstruction of the original sequences using an undamaged homologous sequence or externally supplied donor DNA template to repair the DSBs, while NHEJ repairs the DSBs regardless of homology, leading to insertions or deletions (indels; Hsu *et al*., 2014). In eukaryotic cells, DSBs are preferentially repaired using NHEJ, thus providing a promising editing strategy for research on plant functional genomics and crop improvement (Lieber, 2010; Pan *et al*., 2016). Recently, this system has been successfully utilized to generate mutagenesis in various plant species such as tomatoes, grapes and rice (Brooks *et al*., 2014; Miao *et al*., 2013; Pan *et al*., 2016; Wang *et al*., 2017b), and the mutant allele scan also be stably transmitted to the subsequent generation. Hence, the use of CRISPR/Cas9 to modify the genetic loci associated with ethylene biosynthesis in petunias would hold great promise, even though this technique has not been widely employed in ornamental plants.

In this study, we characterized the expression patterns of the ethylene biosynthesis genes *PhACO1*,* PhACO3* and *PhACO4* in petunia petals at different stages of development. The *PhACO1* gene, which is highly expressed during the flowering period, was edited with the CRISPR/Cas9 system in order to generate *PhACO1*‐edited mutants that exhibited lower ethylene production and subsequently greater flower longevity.

## Results

### Expression patterns of ethylene biosynthesis genes at different flowering stages

**Figure 1:**
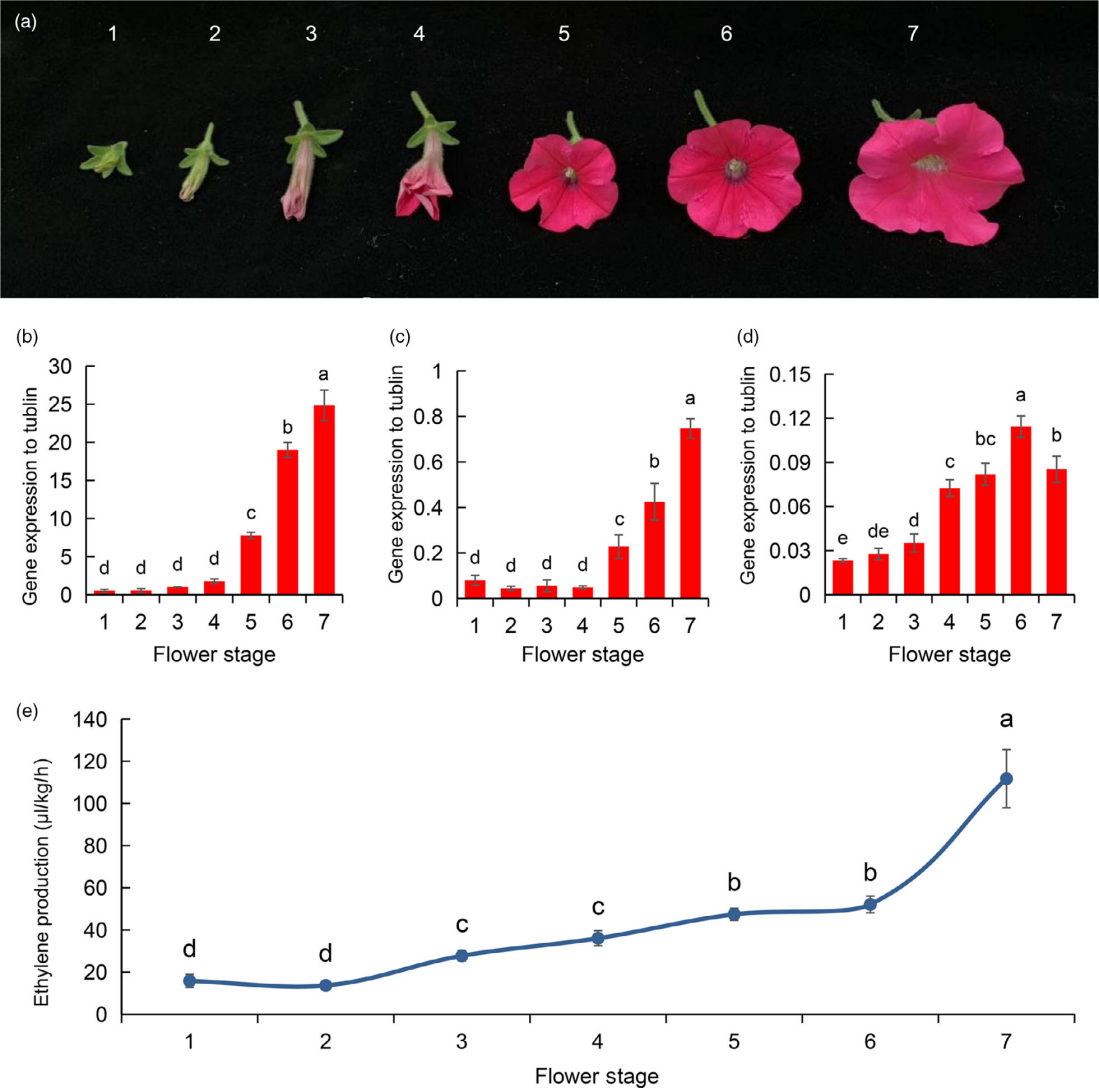
Characterization of the role of ethylene biosynthesis genes in the ethylene production of *Petunia* cv. Mirage Rose:(a) the different flowering stages of *Petunia* cv. Mirage Rose, (b–d) the analysis of expression patterns of the ethylene biosynthesis genes (*PhACO1*,* PhACO3* and *PhACO4*) and (e) the detection of ethylene levels during the different flowering stages.

The differential expression of the ethylene biosynthesis genes (*PhACO1*,* PhACO3* and *PhACO4*) was observed at different flowering stages of *Petunia* cv. Mirage Rose. The expression patterns were similar for *PhACO1* and *PhACO3*; their transcript levels were relatively low during the flower budding stages (stages 1 and 2), started to slightly increase during the early blooming stages (stages 3 and 4) and then continuously increased during the fully blooming stages (stages 5–7; Figure 1b,c). A similar trend was observed for *PhACO4,* but, compared to *PhACO1* and *PhACO3*, higher transcript levels were first observed during the early blooming stage (stage 4) and started to fall during stage 7 (Figure 1d). Generally, the transcript levels of *PhACO1* were higher than those of *PhACO3* and *PhACO4* for all flowering stages (stages 1–7), whereas those of *PhACO4* were the lowest. The ethylene levels detected during the different flowering stages were likely to be associated with the expression patterns of *PhACO1*,* PhACO3* and *PhACO4* because ethylene levels were relatively higher during the blooming stages (stages 5–7) than in the early blooming stages (stages 3 and 4; Figure 1e), with the lowest levels observed during the budding stages (stages 1 and 2).

### *In vivo* validation of the designed Cas9‐sgRNAs for *PhACO1* in petunia protoplasts

**Figure 2:**
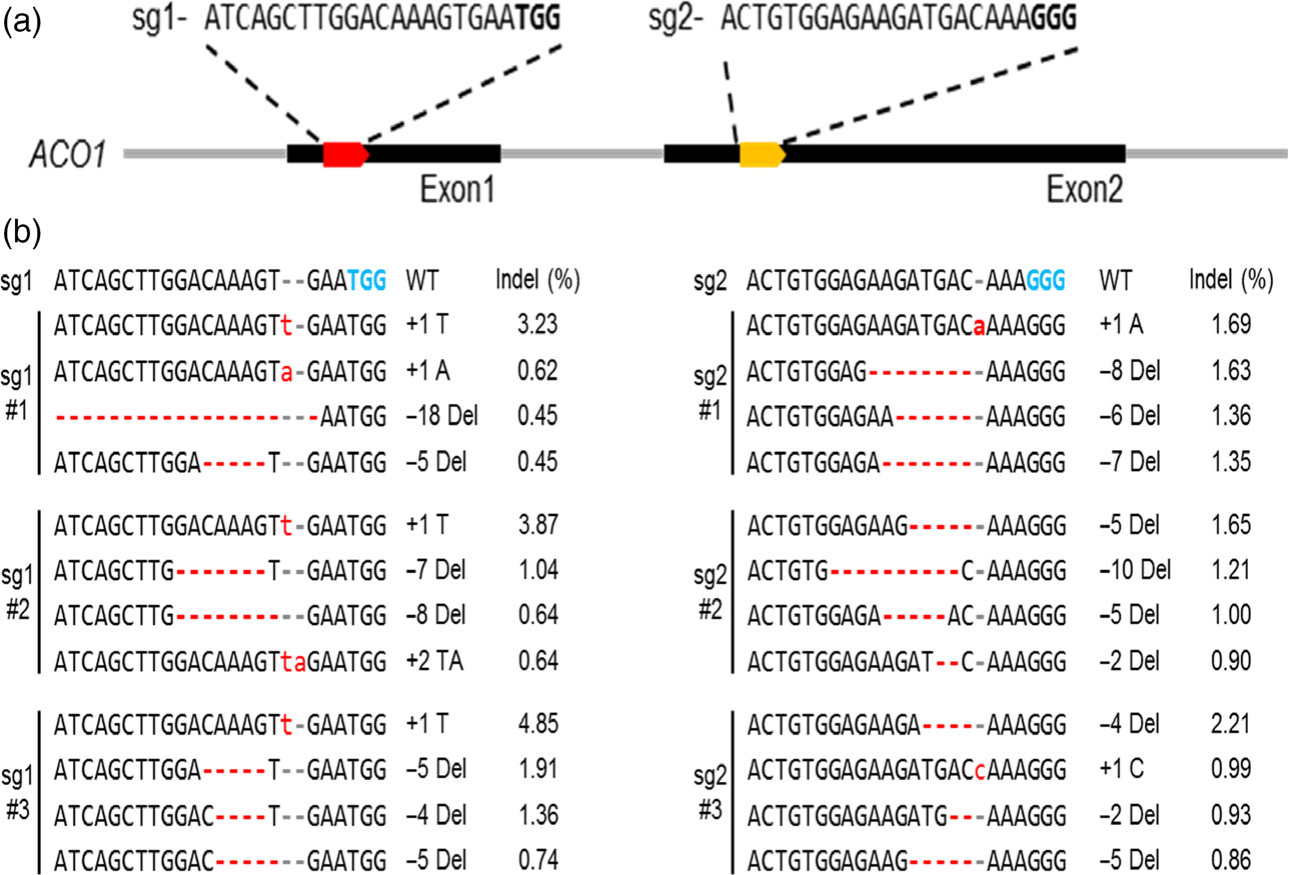
Analysis of *PhACO*_*1*_ gene editing efficiency in petunia protoplasts transfected with the CRISPR/Cas9 RNP complex: (a) generation of different sgRNAs (sgRNA_1_ and sgRNA_2_) from two different target sites (exon 1 and exon 2) in the *PhACO1* genome sequence and (b) illustration of the insertion/deletion (indel) patterns and indel percentages for representative petunia protoplasts transfected with the two different sgRNAs : Cas9. The minus (−) and plus (+) signs indicate the number of nucleotides deleted and inserted at the target sites.

To generate *PhACO1*‐edited petunia plants, we designed two specific sgRNAs using Cas‐Designer equipped in RGEN tools (http://rgenome.net) and CRISPR‐P web tool (http://cbi.hzau.edu.cn/cgi-bin/CRISPR). To assess the genome editing efficiency of designed sgRNAs (sgRNA_1_ and sgRNA_2_) targeting *PhACO1*, petunia protoplasts were transfected with preassembled Cas9–sgRNA ribonuclease protein (RNP) complexes via polyethylene glycol (PEG)‐mediated delivery. After delivering the Cas9 RNP complexes, we incubated the transfected protoplasts at room temperature for 24 h to induce the DSBs at the *PhACO1* loci. Fragments surrounding the targeted sequences of the *PhACO1* were amplified using PCR with the specific primers (Table S2) and analysed with targeted deep sequencing to detect the insertions/deletions (indels) at the expected positions, three base pairs (bp) upstream of an NGG PAM. Mutations were readily detected at all target sites (Figure 2b) after 24 h of incubation, with the indel frequency for sgRNA_1_ observed to be higher than for sgRNA_2_(up to 4.85% and 2.21%, respectively).

### Analysis of the genotype in *PhACO1*‐edited petunias

*PhACO1*, which had the strongest influence of the three target genes on ethylene production in *Petunia* cv. Mirage Rose, was targeted by Cas9 : sgRNA_1_, which exhibited a high indel percentage in protoplast transient assays. The pBAtC : sgRNA_1_ construct was delivered into petunia leaf discs using *Agrobacterium*‐mediated transformation, while discs transformed with an empty vector (pBAtC) were used as the WT. The construct‐transformed leaf discs started to form calluses after a few weeks of culture, and the calluses then produced shoots in phosphinothricin (PPT)‐containing medium. The multiple shoots originating from the callus were regarded as independent transgenic lines. Delivery of the construct in the plants was screened using PCR analysis (Figure S1).

**Figure 3:**
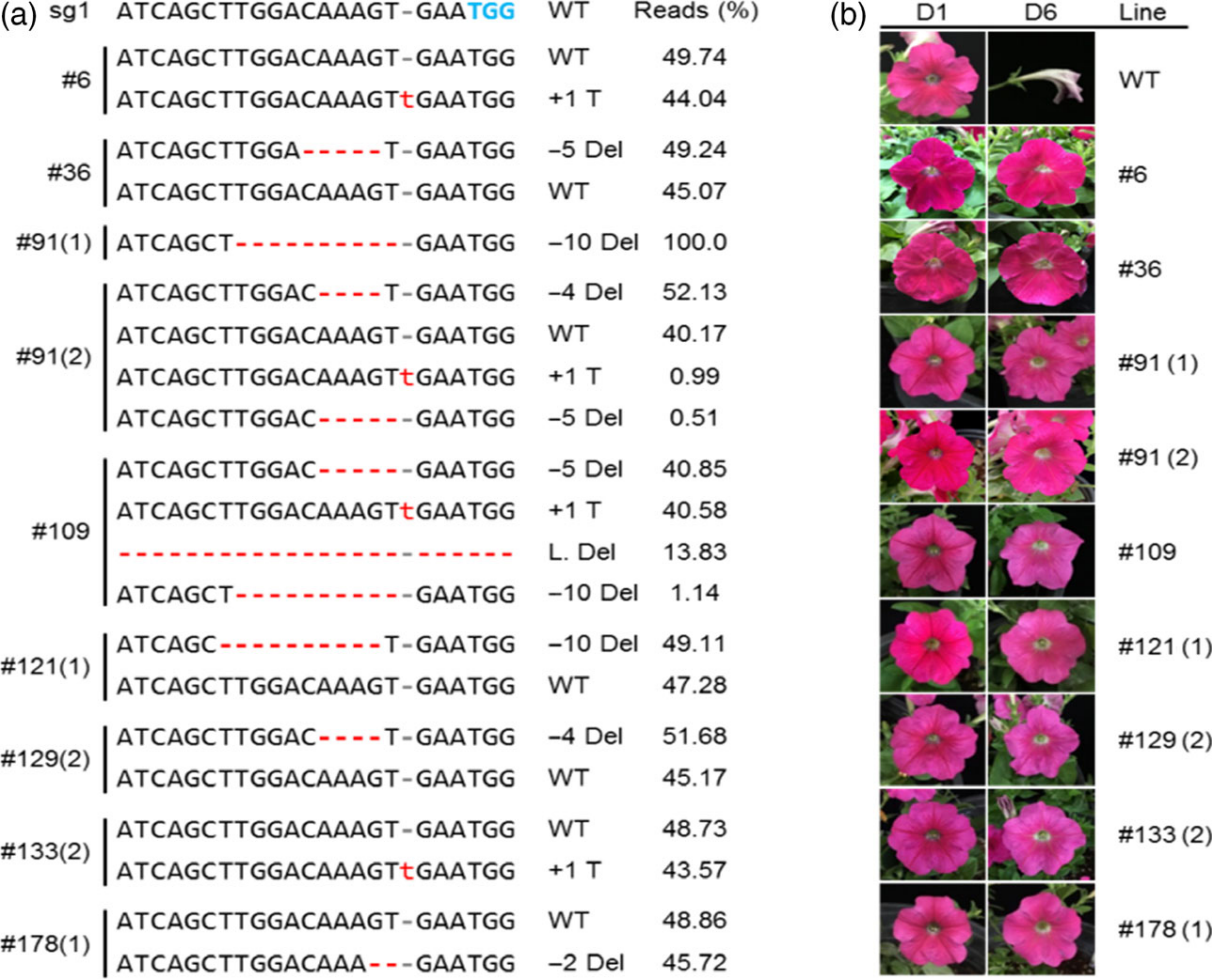
Improvement in flower longevity of *Petunia* cv. Mirage Rose via the editing of the *PhACO1* gene using the CRISPR/Cas9 system:(a) illustration of the insertion/deletion patterns of different *PhACO1*‐edited mutant lines generated by CRISPR/Cas9 : sgRNA_1_ and (b) illustration of the status of flower senescence in the different mutant lines and wild‐type plants on days 1 and 6 after flowering. The minus (−) and plus (+) signs indicate the number of nucleotides deleted and inserted at the target sites.

To evaluate the types of mutation at the *PhACO1* locus of the transgenic plants, PCR amplification and targeted deep sequencing were conducted. Mutant lines showing different indel patterns were observed when pBAtC : sgRNA_1_ was delivered but were not observed in the WT. According to the results shown in Table 1, a mutation frequency of 31.5% was observed following the delivery of pBAtC : sgRNA_1_, with homozygous, monoallelic mutants and chimeric mutants accounting for 2.5%, 15.0% and 82.5% of the mutants, respectively. Homozygous mutants were characterized by a 10‐bp deletion 4 bp upstream of the PAM sites (Figure 3a, line 91[1]), while other mutant lines exhibited different indel patterns at different cleavage sites (Figure 3a). Of the chimeric mutants, we chose lines 91(2) and 109 as representative lines for the determination of flower longevity and ethylene production based on their high indel read percentage. The indel patterns of the other chimeric mutants are presented in Figure S2.

**Table 1:** Percentages of T_0_ plants found with different mutation types in the target sequence

Total examined explants	No. of regenerated shoots	Number of plants with mutation	Mutation frequency (%)	Biallelic/homozygous (%)	Monoallelic (%)	Chimeric (%)
700	127	40	31.5	2.5	15	82.5

### Analysis of the phenotype in *PhACO1*‐edited petunias

**Figure 4:**
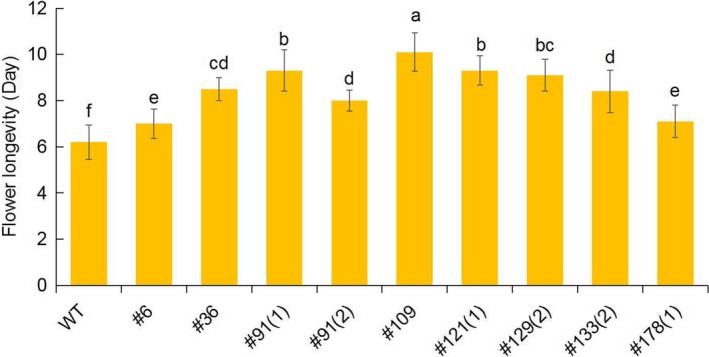
Comparison of the flower longevity for T_0_ mutant lines and wild‐type plants. The data represent the mean of three replicates, and the bars indicate the standard deviation. Means with different letters are statistically significant (*DMRT*,* P < *0.05).

**Figure 5:**
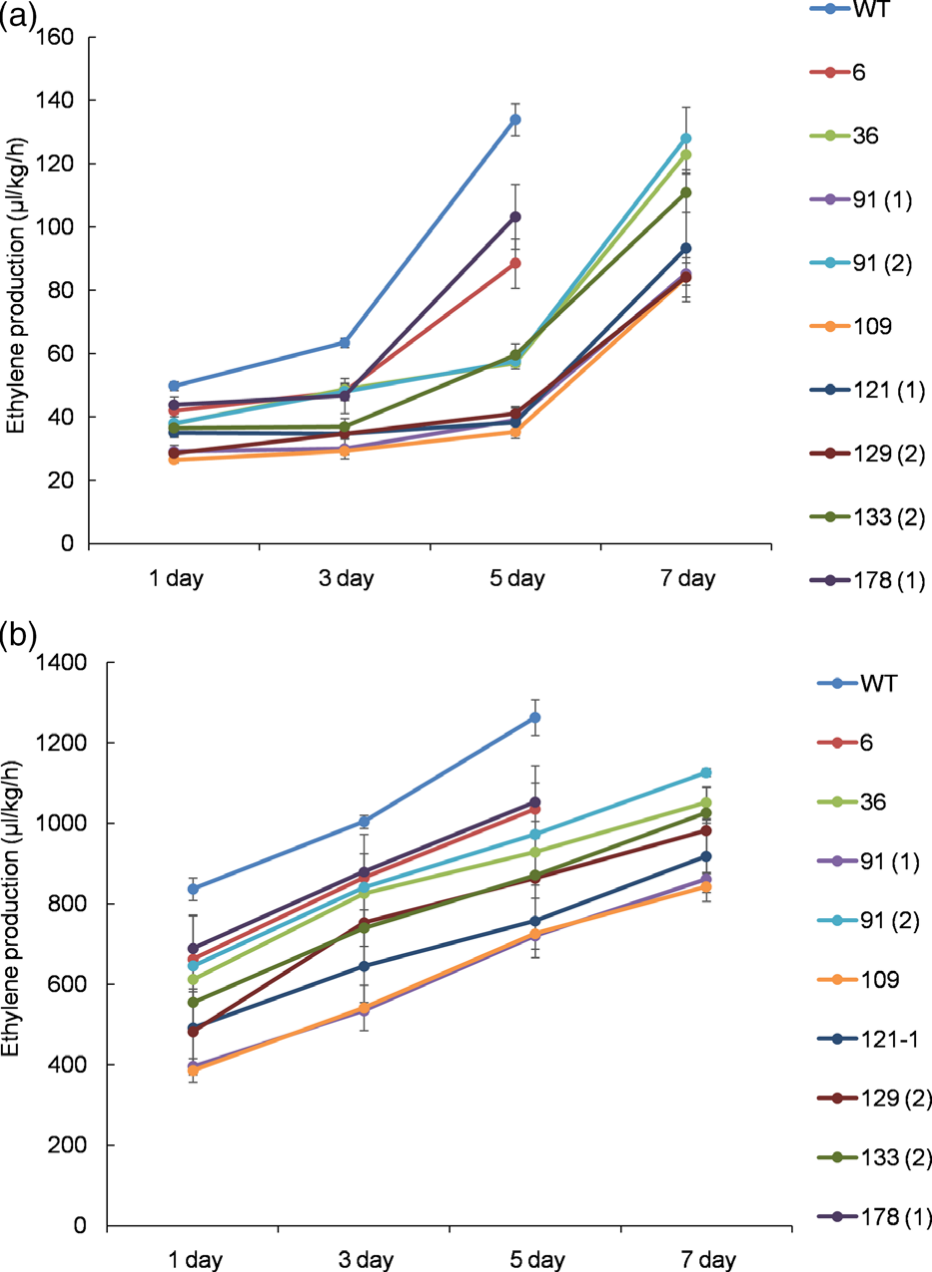
Comparison of ethylene levels detected in the petals (a) and pistil (b) of the T_0_ mutant lines and wild‐type plants on days 1, 3, 5 and 7 after flowering. The data represent the mean of three replicates, and the bars indicate the standard deviation. Means with different letters are statistically significant (*DMRT*,* P < *0.05).

**Figure 6:**
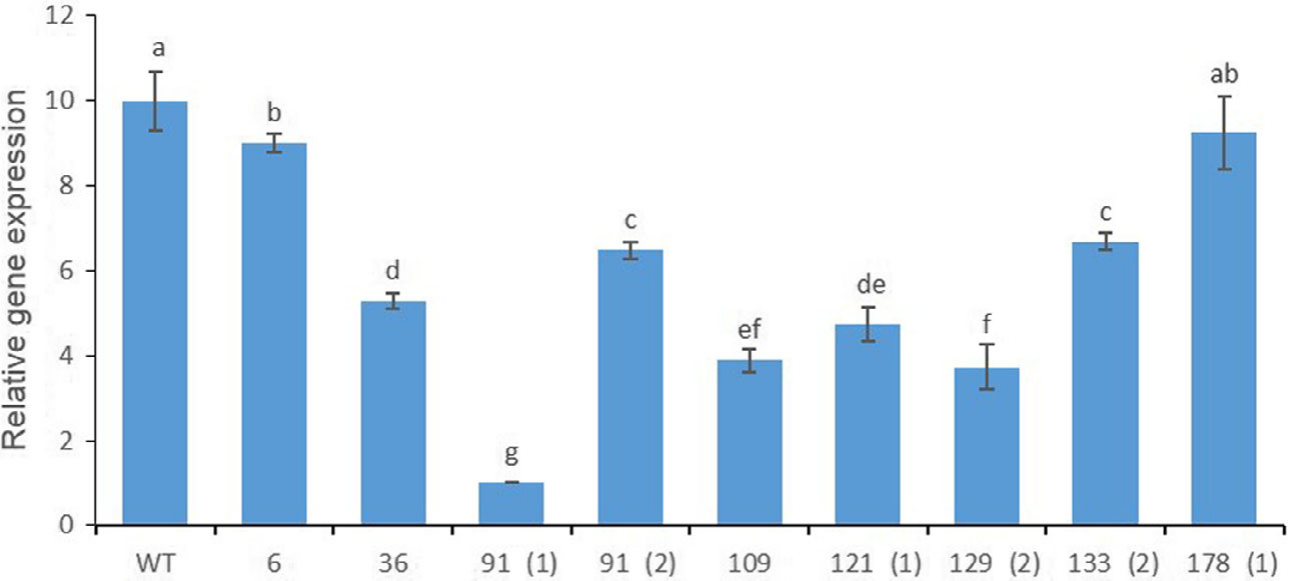
Characterization of the transcript levels for the *PhACO1* gene in CRISPR/Cas9‐mediated *PhACO1*‐edited mutants. The data represent the mean of three replicates, and the bars indicate the standard deviation. Means with different letters are statistically significant (*DMRT*,* P < *0.05).

To determine flower longevity, flowers of the mutant lines, including the WT line, that opened on the same day, were labelled in the mother plants, and they were allowed to bloom until one‐third of the petals exhibited in‐rolling. Of the investigated lines, the flower longevity of line 109 was found to be the highest, followed by lines 91(1), 129(2) and 121(1), while the other lines also exhibited longer flower life than the WT (Figures 3b and 4), which were in accordance with the results for the ethylene levels in the mutant lines. The production of ethylene continuously increased until the end of flowering, though the ethylene levels detected in the WT were notably higher than those of the mutant lines in both the petals and pistils 5 days after full blooming (Figure 5a,b). The lower production of ethylene in lines 109, 121(1), 129(2) and 91(1) was associated with lower transcript levels of the target *PhACO1* gene. In contrast, line 178(1), which exhibited the highest ethylene levels of the mutant lines, did not exhibit significantly lower *PhACO1* transcript levels (Figure 6).

### Evaluation of the potential off‐targets in two homologs, *PhACO3* and *PhACO4*

**Figure 7:**
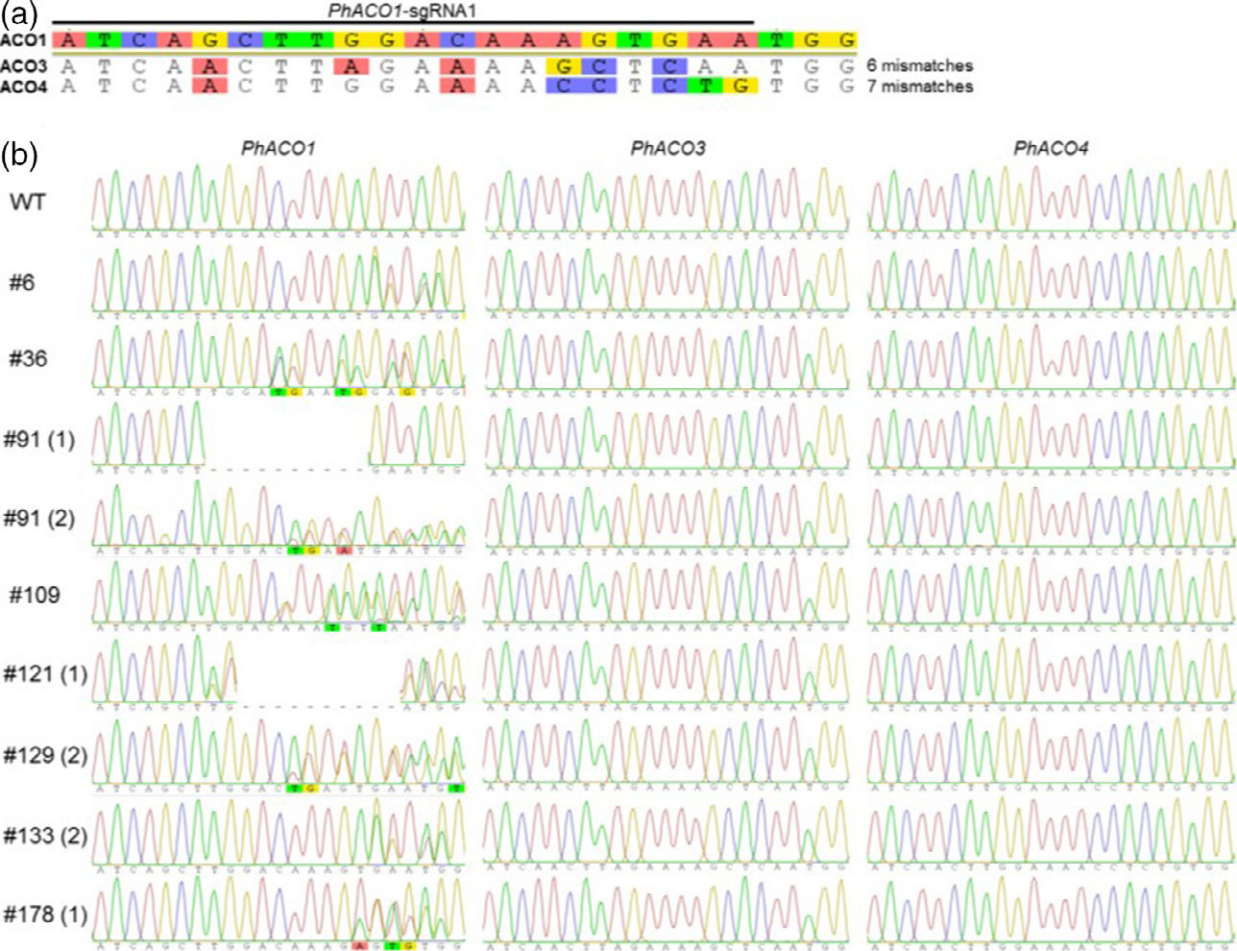
Detection of potential off‐target mutations in CRISPR/Cas9‐mediated *PhACO1*‐edited mutants via Sanger sequencing. (a) Illustration of the number of mismatched nucleotides between the *PhACO1 *: sgRNA1 (on‐target site) and the potential off‐target sites of *PhACO3* and *PhACO4*. (b) Sanger sequencing data showing no mutations at off‐target sites in the *PhACO1*‐edited mutants.

To fully establish that there is a strong association between *PhACO1* gene editing and flower longevity, the off‐target effect on homologous genes *PhACO3* and *PhACO4* was analysed using Sanger sequencing. Compared with the sequence (23 bp) of *PhACO1*‐sgRNA_1_, there were six mismatched regions in *PhACO3* and seven mismatched regions in *PhACO4* (Figure 7a). However, Sanger sequencing revealed that there were no mutations in this region of the homologous genes *PhACO3* and *PhACO4* in any of the *PhACO1*‐edited petunias (Figure 7b), thus proving that there was no off‐target effect.

### Inheritance of *PhACO1* editing in T_1_ petunias

**Figure 8:**
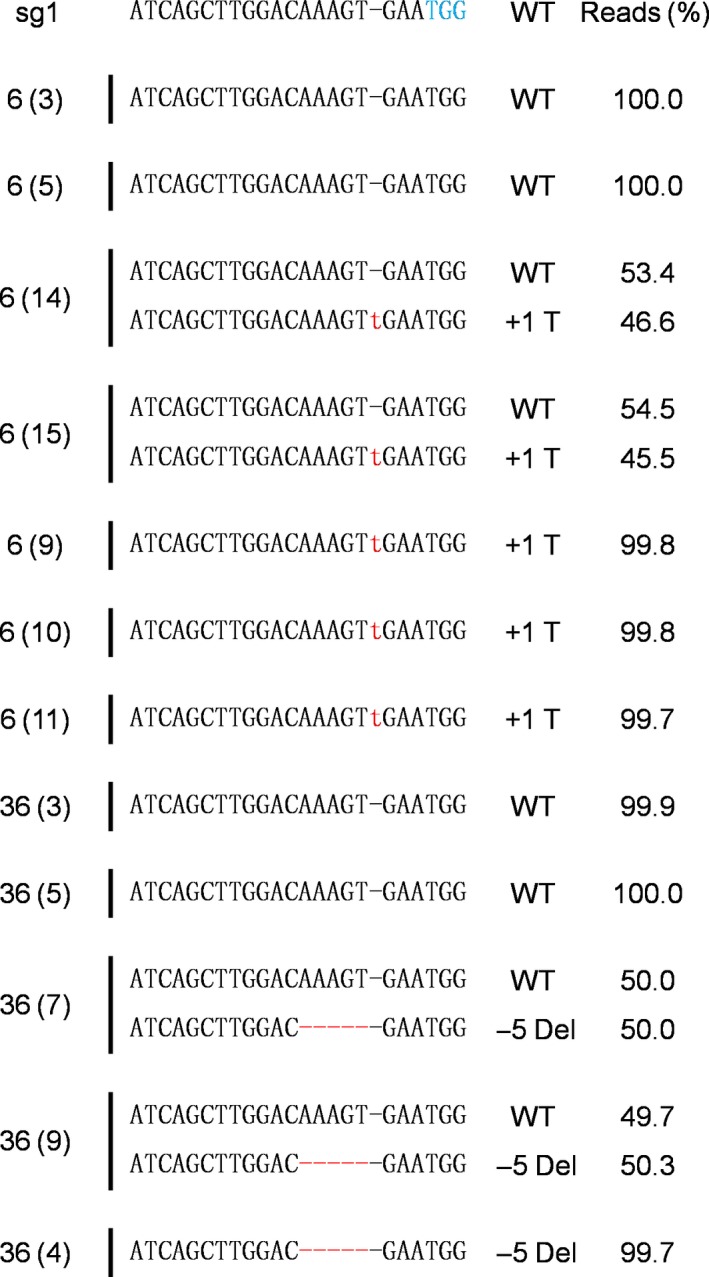
Transmission of the *PhACO1*‐edited gene to T_1_ mutant lines (6 and 36) identified by sequencing. The minus (−) and plus (+) signs indicate the number of nucleotides deleted and inserted at the target sites. WT, wild‐type sequence without mutations at the target sites.

**Figure 9:**
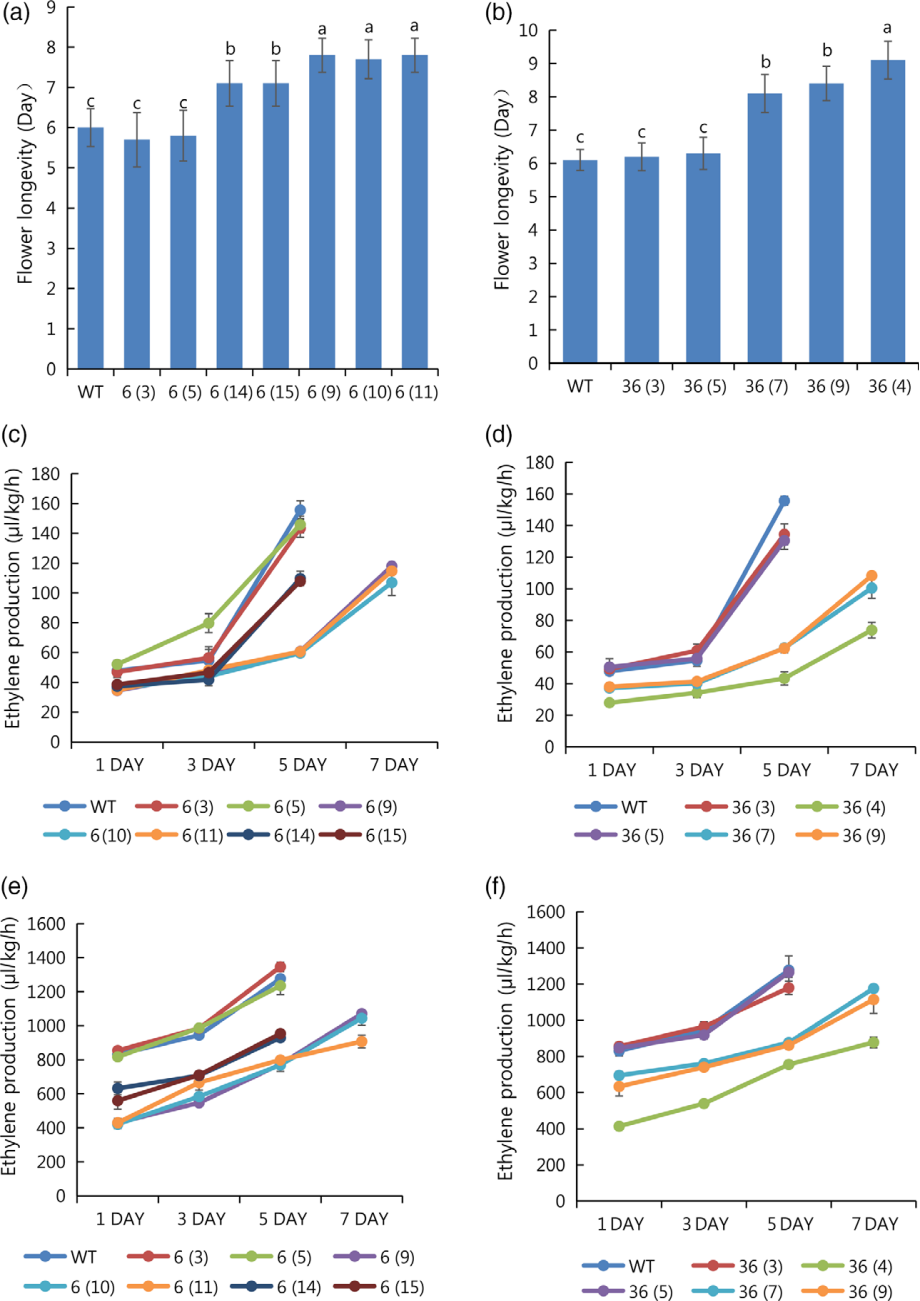
Characterization of flower longevity and ethylene levels in the *PhACO1*‐edited T_1_ mutant lines (6 and 36) and wild‐type (WT) plants: (a, b) flower longevity for different mutant T_1_ lines (6 and 36) and WT specimens, (c, d) ethylene levels measured in the petals of different mutant T_1_ lines (6 and 36) and WT specimens on days 1, 3, 5 and 7 after flowering and (e and f) ethylene levels measured in pistils of different mutant T_1_ lines (6 and 36) and WT specimens on days 1, 3, 5 and 7 after flowering. The data represent the means of three replicates, and the bars indicate the standard deviation. Means with different letters are statistically significant (*DMRT*,* P < *0.05).

T_1_ seedlings of all mutant lines (except line 91‐1) were obtained from self‐pollination in the greenhouse. T_1_ seedlings from the lines were randomly selected and sequenced to analyse sexual transmission, with different patterns of segregation observed in the lines (Table 2). In addition, their indel patterns were also described (Figure 8). For further analysis of flower longevity and ethylene production, T_1_ line 36, consisting of 6% homozygous (1/15), 66% monoallelic (10/15) and 26% WT (4/15), and line 6, consisting of 20% homozygous (3/15), 60% monoallelic (9/15) and 20% WT (3/15), were selected. When flower longevity and ethylene production were evaluated for these lines, the results differed significantly depending on the mutant type. Longer flower longevity was observed in the homozygous mutants, followed by the monoallelic and WT (i.e. homozygous > monoallelic > WT), an outcome that was linked to a reduction in ethylene levels (Figure 9a–f). However, the characteristics of the vegetative and floral organs did not significantly differ between the mutant types (WT, homozygous and monoallelic) of lines 6 and 36 (Table S6). Taken together, it is obvious that the editing of the *PhACO1* gene in *Petunia* cv. Mirage Rose significantly reduced ethylene production and improved flower longevity, and stable results were also observed in *ACO1*‐edited T_1_ seedlings without affecting the morphology of the vegetative and floral organs.

**Table 2:** Segregation patterns of CRISPR/Cas9‐mediated mutagenesis in T_1_ generation

Target gene	Line	T_0_		T_1_	
		Zygosity	Genotype	Mutation segregation	Cas9
ACO1	T_0–6_	Monoallelic	i1WT	3i1i1, 9i1WT, 3WT	All+
	T_0–36_	Monoallelic	d5WT	1d5d5, 10d5WT, 4WT	All+
	T_0–91_ (2)	Chimeric	d4WT	6d4WT	5+;1−
	T_0–109_	Chimeric	i1d5	1i1i1, 2d5d5, 4i1d5, 1i1WT	All+
	T_0–121_ (4)	Monoallelic	d10WT	7d10d10, 6d10WT	All+
	T_0–133_ (2)	Monoallelic	i1WT	1i1i1, 6i1WT, 6WT	All+
	T_0–178_ (1)	Monoallelic	d2WT	5d2d2, 6d2WT, 4WT	10+;5−

+, Cas9 was detected; −, Cas9 was not detected; d#, # of nucleotide(s) deleted at the target sites; i#, # number of nucleotide(s) inserted at target sites; WT, wild‐type sequence without mutations detected at target sites.

## Discussion

The editing of target genes using CRISPR/Cas9 systems has become popular in recent years due to their ability to induce target mutagenesis with more precision and higher efficiency compared to other genome editing technologies. Over the past 5 years, successful genome editing using this technology in plant species such as maize, wheat, tomatoes and rice has been reported (Li *et al*., 2018; Svitashev *et al*., 2016; Zong *et al*., 2017). *Petunia* cv. Mirage Rose (*P. hybrida*) is a bedding plant that is widely used in the landscape industry. As these petunias are typically planted in open landscape fields, the reduction in flower longevity caused by ethylene production cannot be controlled using chemicals that block ethylene biosynthesis. In this study, therefore, we attempted to edit the primary ethylene biosynthesis gene involved in the production of ethylene in *Petunia* cv. Mirage Rose using the CRISPR/Cas9 system.

Initially, we characterized the expression patterns of the ethylene biosynthesis genes *PhACO1*,* PhACO3* and *PhACO4* during different flowering stages (stages 1–7). The transcript levels of the genes were significantly higher during the fully open stages (stages 5–7) than during the initial flowering stages, which are in line with results reported by Tang *et al*. (1994), who observed an increase in ACO mRNA in petunia petals during flower senescence. In the present study, the transcript levels of *PhACO1* in the petals continuously increased and were significantly higher than those of *PhACO3* and *PhACO4*, indicating that the transcript levels of *PhACO1* are more strongly linked to the production of ethylene during the flowering stages. Huang *et al*. (2007) reported the overexpression of the antisense *BoACO1* gene (from broccoli) in petunias, whose sequence is 90% homologous to *PhACO1*, observing the reduction in ethylene production and delayed flower senescence. Therefore, we concluded that *PhACO1* is more closely involved in the production of ethylene in this cultivar than the other two genes.

To edit *PhACO1*, we designed two sgRNAs (sgRNA_1_ and sgRNA_2_) to target *PhACO1*, and transfected petunia protoplasts with a pBAtC : sgRNAs ribonuclease protein (RNP) complex. We found that sgRNA_1_ produced higher indel rates compared with sgRNA_2;_ thus, the former was used to edit *PhACO1*. As expected, a high mutation frequency (31.5%) with different indel patterns and mutant types (i.e. homozygous, monoallelic and chimera) was observed following the delivery of pBAtC : sgRNA_1_ using *Agrobacterium*‐mediated transformation, and the *PhACO1*‐edited mutant lines improved flower longevity compared with WT plants by reducing ethylene levels, regardless of the mutant type. Of the mutant lines, the homozygous line (line 91‐1) and the chimeric line (line 109) significantly reduced the production of ethylene in both petals and pistils, which was linked to the down‐regulation of the target *PhACO1* gene. Moreover, the *PhACO1*‐edited mutants did not have an off‐target effect on two genes (*PhACO3* and *PhACO4*) with a high level of homology, indicating high precision editing of the target gene.

Of the monoallelic mutant lines, lines 121(1) and 129(2) reduced ethylene levels in petals to a greater extent than did the other monoallelic lines (i.e. lines 6, 36, 133[2] and 178[1]). This is linked to the differences in transcript levels for the target *PhACO1* gene among the mutant lines, with lower transcript levels for *PhACO1* in the former group lines than in the latter. It is possible that differences in indel patterns significantly affected the transcript levels of the target gene. From these results, it can be concluded that *PhACO1* is strongly involved in the production of ethylene, which influences flower longevity in *Petunia* cv. Mirage Rose, which is consistent with previous research (Huang *et al*., 2007).

T_1_ petunias inherited most of the mutation types from the T_0_ generation. Similar inheritance patterns have been reported in tomatoes by Pan *et al*. (2016). The results of the present study support the feasibility of using CRISPR/Cas9 to edit a target gene and to transmit the edited gene to the next generation. This contrasts with the lack of homozygotes and biallelic specimens in the T_1_ generation of CRISPR/Cas9 transgenic *Arabidopsis* plants (Feng *et al*., 2014). It is possible that some of the mutations found in somatic cells were lost because the cells did not contribute to germ‐line development (Xu *et al*., 2015). Of the T_1_ mutant lines, ethylene production in the petals and pistils of lines 6 and 36 was analysed. The lowest ethylene production was observed in homozygous mutants, followed by monoallelic and WT for both lines, as observed in the T_0_ plants. In addition, the reduction in ethylene levels was positively associated with an increase in flower longevity for both T_1_ lines (homozygous > monoallelic > WT). These findings also provide further evidence of the stable involvement of *ACO1* in ethylene production in this cultivar. It was also found that editing the *PhACO1* gene in petunias using CRISPR/Cas9 did not affect the morphology of the vegetative and floral organs.

To date, the editing of ethylene biosynthesis genes has not been reported for any plant species; thus, to our knowledge, this is the first report to generate a novel cultivar with extended flower longevity using CRISPR/Cas9 technology. Anthocyanin 1 (*ANT1*; Cermak *et al*., 2015), phytoene desaturase (*SlPDS*) and phytochrome‐interacting factor (*SlPIF4*; Pan *et al*., 2016), which are involved in flavonoid and carotenoid biosynthesis in tomato plants, have been edited using CRISPR/Cas9. In addition, the application of CRISPR/Cas9 in editing the *PDS* gene in petunias has been reported (Zhang *et al*. 2016); however, the editing of the *PDS* gene did not lead to actual improvement in petunia traits because *PDS*‐edited plants are not commercially attractive for the floricultural industry. The present study illustrates that the targeted editing of *PhACO1* can be achieved using CRISPR/Cas9 and that *PhACO1*‐edited petunias exhibit significantly extended flower longevity due to a significant reduction in ethylene production. Hence, we believe that CRISPR/Cas9‐based ethylene tuning is likely to be an effective strategy for enhancing flower longevity in other floricultural crops.

## Conclusion

We investigated the potential roles of *PhACO* genes (*PhACO1*,* PhACO3* and *PhACO4*) in the ethylene production of petunia flowers using transcriptional analysis at different stages of flowering. Of the *PhACO* genes, we revealed that *PhACO1* was most likely to be involved in ethylene biosynthesis. The application of a CRISPR/Cas9 tool in petunias efficiently generated several T_0_ transformants with different in del patterns. The edited petunias exhibited significantly extended flower longevity by reducing ethylene levels compared with the WT. Furthermore, T_1_ petunias inherited the editing patterns, reduced ethylene levels and the phenotype of extended flower longevity that were similar to the parental genotype and phenotype. Our study contributes to a better understanding of the underlying role of *PhACO1* in ethylene biosynthesis and flower longevity. In addition, it provides evidence for the feasibility of the use of CRISPR/Cas9 to precisely edit *PhACO1* and to determine its function; thus, our study can pave the way for the editing of ethylene biosynthesis genes in other ornamental plants, thereby advancing plant biology and the floricultural industry.

## Experimental procedures

### Plant materials

Petunia (*Petunia hybrida* cv. Mirage Rose) seeds obtained from the Hanmi seedling company (Korea America Plug Co., Ltd, Gunpo‐si, Gyeonggi‐do, South Korea ) were sown in plug trays filled with a soil‐less mixture (Berger Co., Quebec, QC, Canada)in a greenhouse with a day/night temperature of 22 °C/18 °C, a photoperiod of 16 h and a relative humidity of 70%. After 2 weeks, the germinated seedlings were transferred to individual pots filled with the same soil and grown under the same greenhouse conditions until flowering.

### Characterization of the expression patterns of ethylene biosynthesis genes

To investigate the expression patterns of ethylene biosynthesis genes (*PhACO1*,* PhACO3* and *PhACO4*) in the flowers of *Petunia* cv. Mirage Rose, petals (approximately 500 mg) from different flowering stages (Figure 1a) were placed in 2‐mL tubes immersed in liquid nitrogen. They were then immediately stored at −80 °C for RNA extraction. Total RNA extraction and reverse transcription were performed as described by Naing *et al*. (2017a). The transcript levels of *PhACO1*,* PhACO3* and *PhACO4* were measured relative to those of alpha‐tubulin gene (reference gene) using the StepOnePlusTM Real‐Time PCR system (Thermo Fisher Scientific, Waltham, MA). Relative gene expression was calculated using the quantitative‐comparative CT (ΔΔ*C*_T_) method. The primers and polymerase chain reaction (PCR) conditions for the detected genes are listed in Table S1. The analysis was repeated three times for each stage.

### Generation of sgRNA and *in vitro* transcription

To perform targeted deletion of *PhACO1* in petunia cv. Mirage Rose, the full complementary DNA (cDNA) sequences of *PhACO1* (GenBank ID L21976.2) were retrieved from NCBI, and the exon–intron boundaries were determined for the gene. We designed two specific sgRNAs using Cas‐Designer in RGEN tools (http://rgenome.net) and the CRISPR‐P web tool (http://cbi.hzau.edu.cn/cgi-bin/CRISPR; Park *et al*., 2015), which contain guide sequences complementary to the different DNA strands of *PhACO1* in the exon regions (exon 1: 19–38 and exon 2: 37–59; Figure 2a).

sgRNAs were synthesized via *in vitro* transcription using T7 RNA polymerase (New England Biolabs, Ipswich, MA) in a reaction buffer (40 mm Tris‐Hcl, 6 mm MgCl2, 10 mm DTT, 10 mm NaCl, 2 mm spermidine, NTPs and RNase inhibitor, pH 7.9) according to the manufacturer's protocol (Kim *et al*., 2014). Transcribed sgRNAs were incubated with DNase I at 37 °C for 30 min to remove template DNA and then purified using PCR purification kits (GeneAll, Seoul Korea).

### Protoplast isolation and transient assays

Protoplasts were isolated from the leaves of 5‐week‐old petunia plants using the valosin‐containing protein (VCP) enzyme (Jie *et al*., 2011). Briefly, the leaves were chopped and digested in 20 mL of enzyme solution at room temperature under gentle shaking (40 rpm) in the dark for 3 h. Following this, the mixture was diluted with an equal volume of W5 solution (154.0 mm NaCl, 125.0 mm CaCl_2_, 5.0 mm KCl, 5.0 mm glucose, 1.5 mm MES) and filtered through a 100‐μm nylon mesh (Falcon, Capitol Scientific, Inc, TX, Austin ) to remove undigested leaf tissue (Yoo *et al*., 2007). Protoplasts were then purified and collected using sucrose gradient centrifugation at 70 ***g*** for 7 min before being transferred to W5 solution (Jie *et al*., 2011).

Recombinant Cas9 protein (30 μg) and *in vitro*‐transcribed sgRNA (80 μg) were mixed and incubated at room temperature for 10 min to generate a ribonuclease protein (RNP) mixture (Woo *et al*., 2015). Protoplasts (1 × 10^5^) in MMG solution (4 mm MES, 0.4 m mannitol and 15 mm MgCl_2_) were then gently mixed with the RNP mixture, and the RNP mixture was transfected into the protoplasts via PEG‐mediated transfection (Woo *et al*., 2015; Yoo *et al*., 2007). Briefly, protoplasts (1 × 10^5^) were blended with the RNP mixture with an equal volume of PEG solution (40% w/v PEG 4000, 0.2 m mannitol and 0.1 m CaCl_2_) for 20 min at room temperature, and then, the protoplasts were washed with W5 solution. The transfected protoplasts were incubated in W5 solution at room temperature for 24 h. After 24 h, the protoplasts were collected by centrifugation and the genomic DNA extracted for analysis using targeted deep sequencing.

### DNA extraction and targeted deep sequencing

Genomic DNA was extracted from transfected protoplasts using the DNeasy Plant Mini Kit (Qiagen, Hilden, Germany). The target region was amplified using nested PCR primer pairs containing adapter sequences. Following this, the amplicons were labelled with an index sequence (Illumina, Seoul, South Korea) using index PCR primer pairs. The targeted deep sequencing of the index PCR amplicons was conducted using an Illumina MiniSeq. The targeted deep sequencing data were analysed using a Cas‐analyzer (Park *et al*., 2016). The site‐specific primer pairs used in this study are listed in Table S2.

### Vector construction

We used a pBAtC vector (accession number KU213971) and Aar I‐mediated sgRNA cloning system (Kim *et al*., 2014) for *Agrobacterium*‐mediated transformation in *Petunia* cv. Mirage Rose. The target sgRNA sequence for *ACO1*‐sg1 was inserted as an annealed oligonucleotide into the AarI‐digested pBAtC vector between the AtU6 promoter and sgRNA scaffold and then circularized by incubation with T4 ligase (New England Biolabs, Ipswich) at room temperature for 3 h. An annealed oligonucleotide product was generated using a mixture containing 100 μmol/L of target oligonucleotides under the following conditions: 95 °C for 5 min, a linear gradient from 95 to 25 °C for 70 min and holding at 10 °C. The *ACO1* (exon 1) targeting sgRNA (gx20) sequence was transcribed under the control of the *Arabidopsis thaliana*‐U6 promoter, and human codon‐optimized *Streptococcus pyogenes* Cas9 (SpCas9) expression was controlled by the 35S‐promoter.

### *Agrobacterium*‐mediated transformation

The transformation of petunia leaf explants was conducted as described by Ai *et al*. (2017). Briefly, approximately 700 leaf explants were excised from 5‐week‐old petunia plants grown in the greenhouse and initially pre‐cultured in are generation medium. The pre‐cultured explants were then infected with *Agrobacterium tumefaciens* that harboured the plasmid vector. The explants were then dried on sterile filter paper and cultured in the regeneration medium in the dark for 2 days. Following this, the explants were transferred to a selection medium containing 0.5 mg/L PPT. Shoots that showed resistance to PPT were transferred to hormone‐free MS medium containing a higher concentration of PPT (1.0 mg/L). Plantlets that produced roots in the PPT‐containing (1.0 mg/L) media were completely removed, washed thoroughly with sterile distilled water, transferred to plastic pots containing peat‐based soil and grown in a greenhouse under controlled conditions.

### DNA extraction, PCR analysis and targeted deep sequencing

Total genomic DNA from the leaves of PPT‐resistant (T_0_) and wild‐type (WT) plants was isolated using the HiYield™ Genomic DNA Mini Kit (plant), as described above. The plasmid pBAtC was used as a positive control, while plants regenerated from explants transformed with the *Agrobacterium* strain (but without sgRNA_1_ : Cas9) were used as WT specimens. PCR was performed using primers, with the PCR conditions described in Table S3. The amplified products were analysed using electrophoresis in 1% (w/v) agarose gels. Targeted deep sequencing for PCR‐positive plants was conducted as described above.

### Determination of the transcript levels of the *PhACO1* gene in the mutants

To determine the transcript levels of the target *PhACO1* gene expressed in the *PhACO1*‐edited mutant lines, total RNA extraction and reverse transcription were performed as described above. In addition, the transcript levels of the genes were also measured as described above. The primer and PCR conditions used for the transcriptional analysis are described in Table S4.

### Determination of flower longevity and ethylene levels

Flowers of the mutant lines, including the WT line, that opened on the same day, were labelled in the mother plants. The labelled flowers were allowed to bloom until one‐third of the petals exhibited in‐rolling, and the longevity of each flower was determined. Longevity was assessed using 10 flowers per mutant line with three replications.

To measure ethylene production, petals and pistils (approximately 500 mg or 50 mg) from each mutant line (including WT) were weighed and sampled 1, 3, 5 and 7 days after flowering. They were placed in a 50‐mL glass tube, which was then sealed with a rubber septum, and ethylene was detected as described by Naing *et al*. (2017a,2017b,2017c). Three replicates were used for each line.

### Sanger sequencing

Total genomic DNA from each T_0_ mutant plant was used as a template to amplify the target regions of the *PhACO* genes by PCR using the corresponding primer pairs (Table S5). Amplicons were directly sequenced using the Sanger sequencing method (Macrogen, Seoul, South Korea).

### Generation of T_1_ lines and the determination of flower longevity and ethylene levels

The flowers from the T_0_ mutant lines were self‐pollinated in the greenhouse. The seeds obtained were then germinated *in vitro* in MS basal medium, and the regenerated plants were analysed using PCR, followed by deep sequencing as described above. Of the T_1_ mutant lines, only lines 6 and 36 were selected to assess flower longevity and ethylene levels using the process described above.

### Statistical analysis

The data were analysed using SPSS v. 11.09 (IBM Corporation, Armonk, NY) and presented as the mean of three replicates. Duncan's multiple range test (DMRT) was used to assess the differences between the means. The significance level was set at *P *<* *0.05.

## Conflicts of interest

There are no conflicts of interest among the authors.

## Author contributions

C. K. K. and A. H. N. designed the experiments. J. ‐S. Kim, B.‐C. K. and H. K. generated all the constructs. J. X analysed transcription levels, flower longevity and ethylene production. B.‐C. K. performed RNP transient assays for the protoplasts. J. X. generated the transgenic lines. B.‐C. K. and S.‐J. B. analysed the Sanger sequencing and targeted deep sequencing. B,‐C. K. helped in writing some parts of the methods section. A.H.N and J. X. statistically analysed all data. A. H. N. wrote the manuscript. A. H. N. and H. K. revised the manuscript. C. K. K supervised the project.

## Supporting information


**Table S1** Primer sequences used for gene expression analysis using quantitative real‐time PCR.
**Table S2** Primer sequences used for nested PCR and 2nd round PCR analysis.
**Table S3** Primers and PCR conditions used for the detection of the presence of the genes using PCR.
**Table S4** Primers used for the transcriptional analysis of *PhACO1* in the mutants.
**Table S5** Primers used for Sanger sequencing.
**Table S6** Comparison of the morphological characteristics of the wild‐type and T_1_ mutant lines.
**Figure S1** Detection of the presence of Cas9‐1, Cas9‐2, and the Basta gene in the transgenic lines in comparison with plasmid and wild‐type specimens using simple PCR.
**Figure S2** Scheme for the insertion/deletion patterns of different T_0_ mutant lines identified by sequencing. Minus (−) and plus (+) signs indicate the number of nucleotides deleted and inserted at the target sites.

## References

[pbi13197-bib-0001] Ai, T.N. , Naing, A.H. , Arun, M. , Jeon, S.M. and Kim, C.K. (2017) Expression of RsMYB1 in petunia enhances anthocyanin production in vegetative and floral tissues. Sci. Hortic. 214, 58–65.

[pbi13197-bib-0002] Brooks, C. , Nekrasov, V. , Lippman, Z.B. and Van Eck, J. (2014) Efficient gene editing in tomato in the first generation using the clustered regularly interspaced short palindromic repeats/CRISPR‐associated9 system. Plant Physiol. 166, 1292–1297. 25225186 10.1104/pp.114.247577 PMC4226363

[pbi13197-bib-0003] Cermak, T. , Baltes, N.J. , Cegan, R. , Zhang, Y. and Voytas, D.F. (2015) High frequency, precise modification of the tomato genome. Genome Biol. 16, 232. 26541286 10.1186/s13059-015-0796-9 PMC4635538

[pbi13197-bib-0004] Cong, L. , Ran, F.A. , Cox, D. , Lin, S. , Barretto, R. , Habib, N. , Hsu, P.D. *et al* (2013) Multiplex genome engineering using CRISPR/Cas systems. Science, 339, 819–823. 23287718 10.1126/science.1231143 PMC3795411

[pbi13197-bib-0005] Feng, Z. , Zhang, B. , Ding, W. , Liu, X. , Yang, D.‐L. , Wei, P. , Cao, F. *et al* (2013) Efficient genome editing in plants using a CRISPR/Cas system. Cell Res. 23, 1229. 23958582 10.1038/cr.2013.114 PMC3790235

[pbi13197-bib-0006] Feng, Z. , Mao, Y. , Xu, N. , Zhang, B. , Wei, P. , Yang, D.L. , Wang, Z. *et al* (2014) Multigeneration analysis reveals the inheritance, specificity, and patterns of CRISPR/Cas‐induced gene modifications in *Arabidopsis* . Proc. Natl Acad. Sci. USA, 111, 4632–4637. 24550464 10.1073/pnas.1400822111 PMC3970504

[pbi13197-bib-0007] Hsu, P.D. , Lander, E.S. and Zhang, F. (2014) Development and applications of CRISPR‐Cas9 for genome engineering. Cell, 157, 1262–1278. 24906146 10.1016/j.cell.2014.05.010 PMC4343198

[pbi13197-bib-0008] Huang, L.C. , Lai, U.L. , Yang, S.F. , Chua, M.J. , Kuo, C.I. , Tsai, M.F. and Sun, C.W. (2007) Delayed flower senescence of Petunia hybrida plants transformed with antisense broccoli ACC synthase and ACC oxidase genes. Postharvest Biol. Technol. 46, 47–53.

[pbi13197-bib-0009] Jie, E.Y. , Kim, S.W. , Jang, H.R. , In, D.S. and Liu, J.R. (2011) Myo‐inositol increases the plating efficiency of protoplast derived from cotyledon of cabbage (*Brassica oleracea var. capitata*). J. Plant Biotechnol. 38, 69–76.

[pbi13197-bib-0010] Kim, J.M. , Kim, D. , Kim, S. and Kim, J.S. (2014) Genotyping with CRISPR‐Cas‐derived RNA‐guided endonucleases. Nat. Commun. 5, 3157. 24445736 10.1038/ncomms4157

[pbi13197-bib-0011] Li, R. , Zhang, L. , Wang, L. , Chen, L. , Zhao, R. , Sheng, J. and Shen, L. (2018) Reduction of tomato‐plant chilling tolerance by CRISPR‐Cas9‐mediated SlCBF1 mutagenesis. J. Agr. Food Chem. 66, 9042–9051. 30096237 10.1021/acs.jafc.8b02177

[pbi13197-bib-0012] Lieber, M.R. (2010) The mechanism of double‐strand DNA break repair by the nonhomologous DNA end‐joining pathway. Ann. Rev. Biochem. 79, 181–211. 20192759 10.1146/annurev.biochem.052308.093131 PMC3079308

[pbi13197-bib-0013] Miao, J. , Guo, D. , Zhang, J. , Huang, Q. , Qin, G. , Zhang, X. , Wan, J. *et al* (2013) Targeted mutagenesis in rice using CRISPR‐Cas system. Cell Res. 23, 1233. 23999856 10.1038/cr.2013.123 PMC3790239

[pbi13197-bib-0014] Naing, A.H. , Lee, K. , Arun, M. , Lim, K.B. and Kim, C.K. (2017a) Characterization of the role of sodium nitroprusside (SNP) involved in long vase life of different carnation cultivars. BMC Plant Biol. 17, 149. 28874121 10.1186/s12870-017-1097-0 PMC5586022

[pbi13197-bib-0015] Naing, A.H. , Win, N.M. , Hang, J.S. , Lim, K.B. and Kim, C.K. (2017b) Role of nano‐silver and the bacterial strain *Enterobacter cloacae* in increasing vase life of cut carnation ‘Omea’. Front. Plant Sci. 8, 1590. 28955374 10.3389/fpls.2017.01590 PMC5601422

[pbi13197-bib-0016] Naing, A.H. , Lee, K. , Kim, K.O. , Ai, T.N. and Kim, C.K. (2017c) Involvement of sodium nitroprusside (SNP) in the mechanism that delays stem bending of different gerbera cultivars. Front. Plant Sci. 8, 2045. 29234346 10.3389/fpls.2017.02045 PMC5712348

[pbi13197-bib-0017] Pan, C. , Ye, L. , Qin, L. , Liu, X. , He, Y. , Wang, J. , Chen, L. *et al* (2016) CRISPR/Cas9‐mediated efficient and heritable targeted mutagenesis in tomato plants in the first and later generations. Sci. Rep. 6, 24765. 27097775 10.1038/srep24765 PMC4838866

[pbi13197-bib-0018] Park, J. , Bae, S. and Kim, J.‐S. (2015) Cas‐Designer: a web‐based tool for choice of CRISPR‐Cas9 target sites. Bioinformatics, 31, 4014–4016. 26358729 10.1093/bioinformatics/btv537

[pbi13197-bib-0019] Park, J. , Lim, K. , Kim, J.‐S. and Bae, S. (2016) Cas‐analyzer: an online tool for assessing genome editing results using NGS data. Bioinformatics, 33, btw561. 10.1093/bioinformatics/btw561 PMC5254075 27559154

[pbi13197-bib-0020] Porat, R. , Borochov, A. and Halevy, A.H. (1993) Enhancement of petunia and dendrobium flower senescence by jasmonic acid methyl ester is via the promotion of ethylene production. Plant Growth Regul. 13, 297–301.

[pbi13197-bib-0021] Reid, M. , Chen, J.‐C. and Jiang, C.‐Z. (2009) Virus‐induced gene silencing for functional characterization of genes in petunia In Petunia (Gerats T. and Strommer J., eds), pp. 381–394. New York, NY: Springer.

[pbi13197-bib-0022] Shan, Q. , Wang, Y. , Li, J. , Zhang, Y. , Chen, K. , Liang, Z. , Zhang, K. *et al* (2013) Targeted genome modification of crop plants using a CRISPR‐Cas system. Nat. Biotechnol. 31, 686–688. 23929338 10.1038/nbt.2650

[pbi13197-bib-0023] Svitashev, S. , Schwartz, C. , Lenderts, B. , Young, J.K. , Mark Cigan, A. (2016) Genome editing in maize directed by CRISPR–Cas9 ribonucleoprotein complexes. Nat. Commun. 7, 13274. 27848933 10.1038/ncomms13274 PMC5116081

[pbi13197-bib-0024] Tang, X. and Woodson, W.R. (1996) Temporal and spatial expression of 1‐aminocyclopropane‐1‐carboxylate oxidase mRNA following pollination of immature and mature petunia flowers. Plant Physiol. 112, 503–511. 12226406 10.1104/pp.112.2.503 PMC157973

[pbi13197-bib-0025] Tang, X. , Wang, H. , Brandt, A.S. and Woodson, W.R. (1993) Organization and structure of the 1‐aminocyclopropane‐1‐carboxylate oxidase gene family from *Petunia hybrida* . Plant Mol. Biol. 23, 1151–1164. 8292780 10.1007/BF00042349

[pbi13197-bib-0026] Tang, X. , Gomes, A.M. , Bhatia, A. and Woodson, W.R. (1994) Pistil specific and ethylene‐regulated expression of 1‐aminocyclopropane‐1‐carboxylate oxidase genes in petunia flowers. Plant Cell, 6, 1227–1239. 12244270 10.1105/tpc.6.9.1227 PMC160515

[pbi13197-bib-0027] Wang, X. , Tu, M. , Wang, D. , Liu, J. , Li, Y. , Li, Z. , Wang, Y. *et al* (2017b) CRISPR/Cas9‐mediated efficient targeted mutagenesis in grape in the first generation. Plant Biotechnol. J. 1–12. 10.1111/pbi.12832 PMC5866948 28905515

[pbi13197-bib-0028] Woltering, E.J. and van Doorn, W.G. .(1988) Role of ethylene in senescence of petals – morphological and taxonomical relationships. J. Exp. Bot. 39, 1605–1616.

[pbi13197-bib-0029] Woo, J.W. , Kim, J. , Kwon, S.I. , Corvalan, C. , Cho, S.W. , Kim, H. , Kim, S.G. *et al* (2015) DNA‐free genome editing in plants with preassembled CRISPRCas9 ribonucleoproteins. Nat. Biotechnol. 33, 1162–1164. 26479191 10.1038/nbt.3389

[pbi13197-bib-0030] Xu, R. , Li, H. , Qin, R. , Li, J. , Qiu, C. , Yang, Y. , Ma, H. *et al* (2015) Generation of inheritable and “transgene clean” targeted genome‐modified rice in later generations using the CRISPR/Cas9 system. Sci. Rep. 5, 11491. 26089199 10.1038/srep11491 PMC5155577

[pbi13197-bib-0031] Yang, S.F. and Hoffman, N.E. (1984) Ethylene biosynthesis and its regulation in higher‐plants. Annu. Rev. Plant Physiol. Plant Mol. Biol. 35, 155–189.

[pbi13197-bib-0032] Yoo, S.D. , Cho, Y.H. and Sheen, J. (2007) *Arabidopsis* mesophyll protoplasts: a versatile cell system for transient gene expression analysis. Nat. Protoc. 2, 1565–1572. 17585298 10.1038/nprot.2007.199

[pbi13197-bib-0033] Zhang, B. , Yang, X. , Yang, C. , Li, M. and Guo, Y. .(2016) Exploiting the CRISPR/Cas9 system for targeted genome mutagenesis in Petunia. Sci Rep 6, 20315. 26837606 10.1038/srep20315 PMC4738242

[pbi13197-bib-0034] Zong, Y. , Wang, Y. , Li, C. , Zhang, R. , Chen, K. , Ran, Y. , Qiu, J.L. *et al* (2017) Precise base editing in rice, wheat and maize with a Cas9‐cytidine deaminase fusion. Nat. Biotechnol. 35, 438–440. 28244994 10.1038/nbt.3811

