# Supplementary material for: CRISPR/Cas9‐mediated editing of 1‐aminocyclopropane‐1‐carboxylate oxidase1 enhances Petunia flower longevity
Source: Plant Biotechnol J. 2019 Jul 2;18(1):287–97. doi: 10.1111/pbi.13197 (PMC6920161; doi:10.1111/pbi.13197)
Supplement: Supplementary file 1 — Table S1 Primer sequences used for gene expression analysis using quantitative real‐time PCR. Table S2 Primer sequences used for nested PCR and 2nd round PCR analysis. Table S3 Primers and PCR conditions used for the detection of the presence of the genes using PCR. Table S4 Primers used for the transcriptional analysis of PhACO1 in the mutants. Table S5 Primers used for Sanger sequencing. Table S6 Comparison of the morphological characteristics of the wild‐type and T1 mutant lines. Figure S1 Detection of the presence of Cas9‐1, Cas9‐2, and the Basta gene in the transgenic lines in comparison with plasmid and wild‐type specimens using simple PCR. Figure S2 Scheme for the insertion/deletion patterns of different T0 mutant lines identified by sequencing. Minus (−) and plus (+) signs indicate the number of nucleotides deleted and inserted at the target sites. [file PBI-18-287-s001.docx]

**Supporting Information Materials**

**Supplementary Table 1.** Primer sequences used for gene expression analysis using quantitative real-time PCR

| Gene (Accession No.) | | Primers sequences (5'-3') | |
| --- | --- | --- | --- |
|  |  | Forward primer | Reverse primer |
| *PhACO1* | L21976.2 | 5'- ATCAGCTTGGACAAAGTGAATGG-3' | 5'-CACCAACTCAAAGAAGCCCC-3' |
| *PhACO3* | L21978.1 | 5'-GAGTGGCCTCCAACTACTAAAAG-3' | 5'-CATTAGTGATCACCTCAAGTTGGT-3' |
| *PhACO4* | L21979.1 | 5'-ACTTGAGGTGATCACTAACGG-3' | 5'-ACCTTGGCTCTTTTGCCTGA-3' |
| *Tubulin* | SGN-U207876 | 5'-TGGAAACTCAACCTCCATCCA-3' | 5'-TTTCGTCCATTCCTTCACCTG-3' |

*TUB: tubulin gene (Mallona et al., 2010).

*PhACO1*, *Petunia x hybrida* 1-aminocyclopropane-1-carboxylate oxidase (ACO1); *PhACO3*, *Petunia x hybrida* 1-aminocyclopropane-1-carboxylate oxidase (ACO3); *PhACO4*, *Petunia x hybrida* 1aminocyclopropane-1-carboxylate oxidase (ACO4). PCR conditions: 95℃ for 10 min followed by 40 cycles of 95℃ for 30 s, 60℃for 30 s, 95℃for 15 s, 60℃ for30 s, and 95℃for 15 s.

**Supplementary Table 2.** Primer sequences used for nested PCR and 2^nd^ round PCR analysis

| **PCR** | **Primer sequences** (5'-3') |
| --- | --- |
| **Nested PCR** | **F- TCGACTTGTATTACTTGTTGGCC**  **R- GCAAGCTTACATACACCTCGA** |
| **2^nd^ round PCR (sgRNA1)** | **F- ACACTCTTTCCCTACACGACGCTCTTCCGATCTACATTCATCTTCTGTTATTCACACA**  **R-GACTGGAGTTCAGACGTGTGCTCTTCCGATCTTTAATTACCTCAAAGAAGCCCCAG** |
| **2^nd^ round PCR (sgRNA2)** | **F-ACACTCTTTCCCTACACGACGCTCTTCCGATCTTTTGAGGTAATTAACTTTCTTCACGT**  **R- GACTGGAGTTCAGACGTGTGCTCTTCCGATCTAAAGGTGCTTTCCCAATCCA** |

**Supplementary Table 3.** Primers and PCR conditions used for detection of the presence of the genes using PCR analysis

| Genes | Primer sequences | PCR condition |
| --- | --- | --- |
| *Bar* | FP: 5'-TCAGATTTCGGTGACGGGCA-3' | 95 ◦C for 2 min, followed by 35 cycles of 95 ◦C for 20 s, 60 ◦C for 30 s, 72 ◦C for 30 s, and 72 ◦C for 5 min |
|  | RP: 5'-ATGAGCCCAGAACGACGC-3' |  |
| *Cas9 1* | FP: 5'-CCCACGAGGAGCATCGTGG-3' | 95 ◦C for 2 min, followed by 35 cycles of 95 ◦C for 20 s, 61 ◦C for 40 s, 72 ◦C for 1 min, and 72 ◦C for 5 min |
|  | RP: 5'-TGCTGAAGATCTCCTGCAGG-3' |  |
| *Cas9 2* | FP: 5'-CAGGCCGAGAACATCATCC-3' | 95 ◦C for 2 min, followed by 35 cycles of 95 ◦C for 20 s, 61 ◦C for 40 s, 72 ◦C for 1 min, and 72 ◦C for 5 min |
|  | RP: 5'-GCATGCCTGCAGGTCACTGG-3' |  |

**Supplementary Table 4.** Primer used for transcriptional analysis of *PhACO1* in the mutants

| Target gene | Accession No. | Primers sequences (5'-3') | |
| --- | --- | --- | --- |
|  |  | Forward | Reverse |
| *ACO1* | L21976.2 | GGTGTTGAAAGAGCTGCCACTA | CTCAAAGAAGCCCCAGTTTTCAC |
| Tubulin | SGN-U207876 | TGGAAACTCAACCTCCATCCA | TTTCGTCCATTCCTTCACCTG |

**TUB: tubulin gene (Mallona et al., 2010)*

PCR conditions: 95℃ for 10 min followed by 40 cycles of 95℃ for 30 s, 60℃for 30 s, 95℃for 15 s, 60℃ for30 s, and 95℃for 15 s.

**Supplementary Table 5.** Primer sequences used for Sanger sequencing

| **Genes** | **Primer sequences** (5'-3') |
| --- | --- |
| ***PhACO1*** | F- TCGACTTGTATTACTTGTTGGCC  R- GAGGCACATGGAACATCTT |
| ***PhACO3*** | F- ACCCAAGAAAGCCTCACTCT  R- TCCAACTGATACACTGACATCT |
| ***PhACO4*** | F- AGCAAGAACACACCTACTTTCT  R- TCTCAACATCTAATTCTGGCAGT |

**Supplementary Table 6.** Comparison of morphological characteristics of WT and T_1_ mutant lines

Data represent the mean of three replicates, and the bar indicates the standard deviation. Means with different letters are statistically significant (*DMRT*, *P <* 0.05).

**Supplementary Figures**

**Supplementary Fig. 1.** Detection of the presence of Cas9-1, Cas9-2 and bar gene in the transgenic lines in comparison with plasmid (P) and wild type (WT) using simple PCR


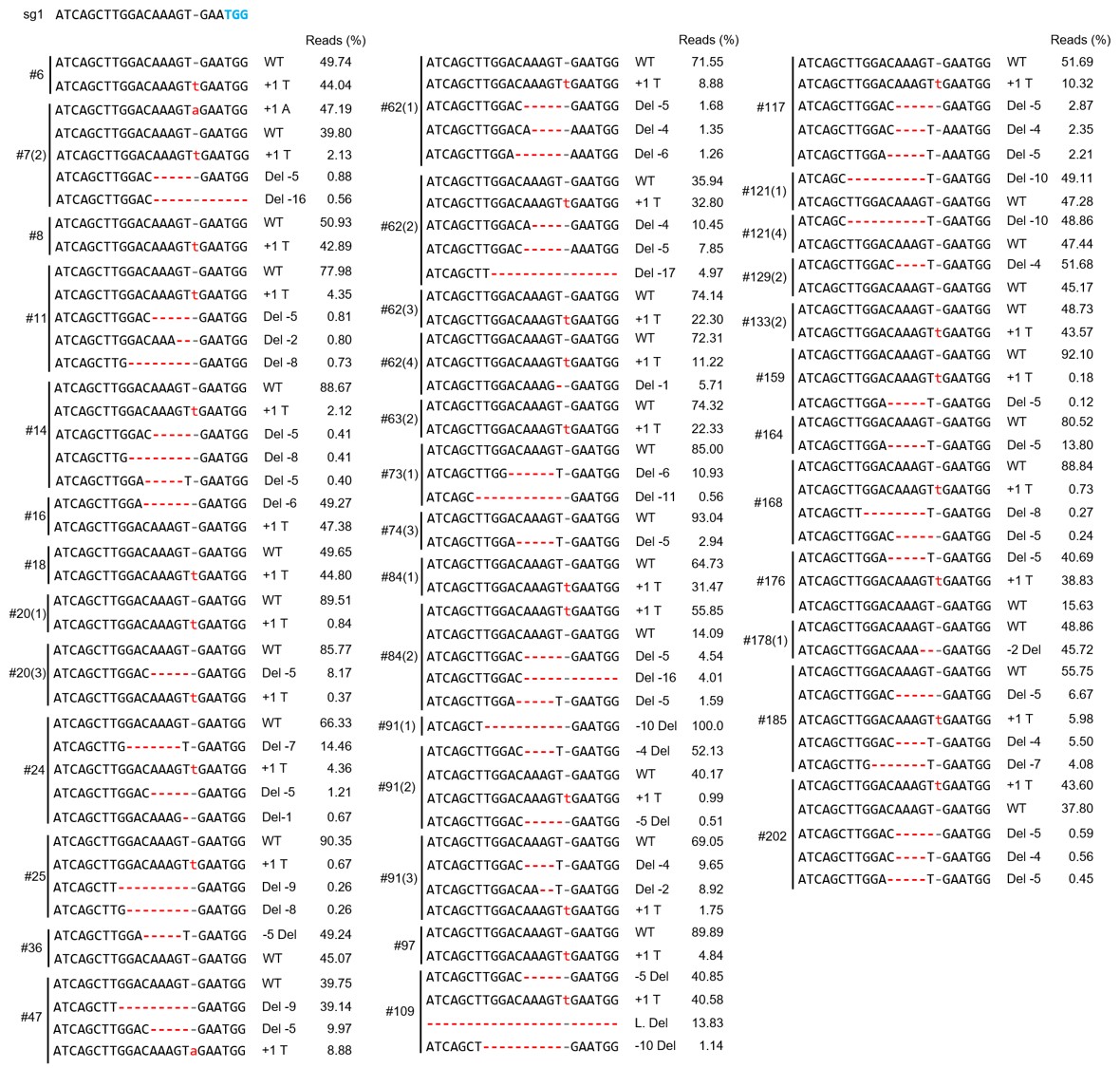


**Supplementary Fig. 2.** Scheme of insertion/deletion patterns of different T_0_ mutant lines identified by sequencing. Minus (–) and plus (+) signs indicate the number of nucleotides deleted and inserted at the target sites.
